# Long-Term Stochasticity Combines With Short-Term Variability in Assembly Processes to Underlie Rice Paddy Sustainability

**DOI:** 10.3389/fmicb.2020.00873

**Published:** 2020-05-15

**Authors:** Wenjing Liu, Emily B. Graham, Linghao Zhong, Jianwei Zhang, Shijie Li, Xiangui Lin, Youzhi Feng

**Affiliations:** ^1^State Key Laboratory of Soil and Sustainable Agriculture, Institute of Soil Science, Chinese Academy of Sciences, Nanjing, China; ^2^The College of Resources and Environment, University of Chinese Academy of Sciences, Beijing, China; ^3^Pacific Northwest National Laboratory, Richland, WA, United States; ^4^Department of Chemistry, Pennsylvania State University, Mont Alto, PA, United States; ^5^State Key Laboratory of Environmental Geochemistry, Institute of Geochemistry, Chinese Academy of Sciences, Guiyang, China; ^6^State Key Laboratory of Lake Science and Environment, Nanjing Institute of Geography and Limnology, Chinese Academy of Sciences, Nanjing, China

**Keywords:** paddy soil, neutral theory, niche theory, microbial ecology, community ecology, null model

## Abstract

Revealing temporal patterns of community assembly processes is important for understanding how microorganisms underlie the sustainability of agroecosystem. The ancient terraced rice paddies at Longji provide an ideal platform to study temporal dynamics of agroecosystem sustainability due to their chronosequential records of soil physicochemistry and well-archived microbial information along 630-year rice cultivation. We used statistical null models to evaluate microbial assembly processes along the soil chronosequences of Longji rice paddies through time. Stochastic and deterministic assembly processes jointly governed microbial community composition within successional eras (less than 250 years), and within-era determinism was mainly driven by soil fertility and redox conditions alone or in combination. Conversely, across successional eras (i.e., over 300 years), stochasticity linearly increased with increasing duration between eras and was eventually predominant for the whole 630 years. We suggest that the impact of stochasticity vs. determinism on assembly is timescale-dependent, and we propose that the importance of stochastic assembly of microbial community at longer timescales is due to the gradual changes in soil properties under long-term rice cultivation, which in turn contribute to the sustainability of paddy ecosystem by maintaining a diverse community of microorganisms with multi-functional traits. In total, our results indicate that knowledge on the timescales at which assembly processes govern microbial community composition is key to understanding the ecological mechanisms generating agroecosystem sustainability.

## Introduction

Agroecosystem sustainability can be defined as meeting society’s current needs for food without compromising the needs of future generations (Tilman et al., 2011; Liu et al., 2015a). Soil fertility is the basis of sustainability in agroecosystems because it is central in maintaining crop productivity while minimizing the need for fertilizer application that has deleterious effects on environmental and human health (Doran and Zeiss, 2000; Wall et al., 2015; Rinot et al., 2019). Microbes play critical roles in soil fertility due to their contribution to, for example, organic matter decomposition, nutrient cycling and contaminant pools. Thus, it soil microbial communities should have direct implications for sustainability of crop yields (van der Heijden et al., 2008; Hall et al., 2018). Larger numbers of microbial species tend to encompass a broader variety of functional traits that support these ecological functions (Cardinale et al., 2006; Hector and Bagchi, 2007; Pennekamp et al., 2018). Thus, scientists conceptually link higher microbial diversity and function to greater agroecosystem sustainability (Cardinale et al., 2002; Bell et al., 2005; Patsch et al., 2018).

Assembly processes govern temporal patterns in microbial diversity during the development of a given ecosystem, which in turn influence its ecological function and broader ecosystem sustainability (Knelman and Nemergut, 2014). Understanding temporal patterns of succession and assembly processes would help to understand and even manage sustainability of ecosystems. For these reasons, a vast body of investigations has explored relationships between soil pedogenesis and microbial ecology, including salt marshes (Dini-Andreote et al., 2014) and glacial landscapes (Castle et al., 2016). Furthermore, many researchers have used the framework of community assembly developed by Vellend (2010) and modified by Stegen et al. (2013), which well disentangles the contributions of four ecological processes [homogenizing dispersal and dispersal limitation plus drift (stochastic assembly) vs. variable and homogeneous selections (deterministic assembly)] to explore mechanisms driving microbial community composition along temporal chronosequences (Dini-Andreote et al., 2015; Graham et al., 2016; Yan et al., 2017). For example, deterministic processes have often been shown to be the dominant factor governing microbial compositional turnover along temporal gradients (Vanwonterghem et al., 2014; Tripathi et al., 2018). Stochastic processes have been recently identified as another important driver for microbial community composition (Chase, 2010; Zhou and Ning, 2017), and stochastic assembly of microbial community has been suggested to foster high rates of ecosystem functioning by maintaining biodiversity (Graham and Stegen, 2017). In addition, deterministic and stochastic processes govern temporal community assembly in unison (Nemergut et al., 2013; Zhou and Ning, 2017). Yet, while many studies have investigated microbial community assembly within the framework of our contemporary (or short-term) environment, we still lack knowledge on how assembly processes shift over decadal-to-centurial timescales that are vital in making accurate predictions of ecosystem sustainability.

Two conceptual models have been suggested to explain temporal patterns in community assembly. One emphasizes that environmental (or niche) variations due to historical events are the main driving force mediating the balance of ecological assembly processes. This model proposes that environmental changes through short-term succession, e.g., several months, induce fluctuations in the strength of stochastic vs. deterministic assembly processes (Ferrenberg et al., 2013). Another model suggests that time itself acts as a driver of community assembly over longer timescales (e.g., around 100 years), for example, by inducing shifts from stochastic to deterministic process (Dini-Andreote et al., 2015) or vice versa (Brown and Jumpponen, 2015) due to the aggregation of dispersal events or selective pressures. As more and more of these events accumulate through time, the community moves towards a more stochastic or deterministic assemblage. Both models reflect the notion that the balance of stochastic and deterministic processes is time dependent (Zhang et al., 2016; Tripathi et al., 2018), but the directionality of and extent to which changes in environmental variables through time shift the balance of deterministic and stochastic processes is unknown (Dini-Andreote et al., 2015; Graham et al., 2016, 2017).

Flooded rice fields are agroecosystems with high level of sustainability (Zhang and Gong, 2003; Zhu et al., 2016). Microbial succession during pedogenesis and development of paddy soils have been extensively investigated, commonly by using a space-for-time substitution approach (Zou et al., 2015; Ding et al., 2017). Space-for-time substitutions use a well-characterized age gradient, but the impacts of other state factors (i.e., atmospheric conditions, parent material, surface biodiversity) are typically not considered. Alternatively, soil profile chronosequences that are formed from the same parent in the same climate conditions encompass knowledge on soil pedogenesis and development (Chen et al., 2014). The 630-year old Longji terraced paddy soil has a clear chronosequence with orderly changes in physicochemistry (Jiang et al., 2014) that are closely associated with variation in microbial community composition (Feng et al., 2017a; Jiang et al., 2017). Longji chronosequences have also undergone changes in soil fertility and transitions between anoxic and oxic status, due to anthropogenic activity and palaeoclimate change. Collectively, these features make them an ideal platform to investigate temporal microbial community assembly and to explore the key processes maintaining sustainable agroecosystems.

Both Stegen et al. (2012); Tripathi et al. (2018) posited that deterministic processes occur under drastic and/or sudden variation in environment conditions, whereas stochastic assembly is dominant when there are mild and/or gradual changes. Paddy soils are characterized by consistent flooding which tempers extremes in environmental changes and in responses to external perturbation. Flooding also increases hydrologic connectivity that putatively increases stochastic dispersal. Therefore, we hypothesized that the stochastic processes dominated assembly in general and that these processes were central to maintaining microbial diversity during succession across the 630-year rice paddy chronosequences. We also hypothesized that the balance of stochastic and deterministic ecological processes would fluctuate within successional eras depending on the prevailing environment. To test our hypotheses, we investigated the assembly processes governing bacterial community composition through successional stages across 630-year period. Our investigation elucidates both long- and short-term mechanisms governing microbial community that are essential for managing sustainable agrosystems in the face of long-term environmental change.

## Materials and Methods

### Site Description and Soil Sampling

The Longji rice terraces are located in Longsheng county of Guilin city in Guangxi autonomous region, and experience an annual average temperature of 14.4−16.9°C and annual precipitation of 1600−1733 mm. Structurally, Longji rice terraces are generally parallel to the contours of the hillside, consisting of flat treads (cultivated areas), terrace walls (risers), and field ridges on the top of the risers. With long-term rice cultivation, the plow layer of terraces has gradually turned into plow pan, a densified soil layer with low infiltration, making the cultivated horizon a bottom-up chronological sedimentary sequence whose soil physiochemical properties reflect historical information (Jiang et al., 2014; Jiang et al., 2017). The soil profile LJTT-3 (800 m asl, 25°45′ 4061′ N, 110°6.99′ E) was chosen for our investigation because of the long chronosequence (∼630 year) of this cultivated horizon. Soil chemical properties were analyzed according to the protocols of Lu (1999). Detailed information on soil chemical properties was provided in Supplementary Figure S1. Radiocarbon ages were evaluated by AMS ^14^C dating in accelerator Mass Spectrometry Laboratory of Peking University. Then, ages were calibrated using the OxCal V4.1.7 program (Ramsey, 2009) and the IntCal09 calibration curve (Reimer et al., 2009), and the calibrated ages were determined by weighted averaging of ages at 2σ precision. A total of 69 [23 layers (e.g., 1, 3, 5, …, 45 cm) × triplicate] samples were taken from profile LJTT-3 at intervals of 2 cm vertical intervals from 0 to 45 cm depth spanning 630-year of rice cultivation (ranging from 2000 to 1384 year). All the samples were stored in −40°C for microbial assays.

### Soil DNA Extraction

DNA was extracted from a total of 69 composite soil samples using the FastDNA SPIN Kit for soil (MP Biomedicals, Santa Ana, CA, United States) according to the manufacturer’s instructions. The extracted DNA was dissolved in 50 μL of TE buffer, and was qualified by gel electrophoresis. After that, the extracted DNA was evaluated by NanoDrop 2000 (Themo Fisher Scientific, United States) for quantity and quality and stored at −20°C until further usage.

### PCR and Processing of the High-Throughput Sequencing Data

PCR amplification was conducted for the bacterial community with primer set 519F (3′-CAGCMGCCGCGGTAATWC-5′) and 907R (3′-CCGTCAATTCMTTTRAGTTT-5′) targeting the V4-V5 region of the bacterial 16S rRNA gene. The 5-bp bar-coded oligonucleotides were fused to the forward primer. Each PCR 50 μL reaction mixture contained 1.25 μM of deoxynucleoside triphosphate, 2 μL (15 μM) of forward and reverse primers, 2 μM of Taq DNA polymerase (TaKaRa, Japan), and 1 μL (50 ng) of genomic community DNA as a template. Each reaction was performed in triplicate to counter biases generated during amplification. The negative control was always run with sterile water as the template instead of soil DNA extract. Amplifications were carried out using the thermal conditions as follows: 94°C for 5 min, 30 cycles (94°C for 30 s, 55°C for 30 s, and 72°C for 45 s), and a final extension at 72°C for 10 min. Reaction products for each sample were pooled and purified using the QIAquick PCR Purification Kit (Qiagen), and quantified using NanoDrop ND-2000 (Thermo Fisher Scientific, United States).

High-throughput sequencing was performed with Illumina Hiseq sequencing platform (Illumina Inc.). The bar-coded PCR products from all samples were normalized in equimolar amounts before sequencing. After sequencing, 16S rRNA genes data were processed using the Quantitative Insights Into Microbial Ecology (QIIME, United States) pipeline for data sets. Sequences with a quality score below 20 and the length fewer than 200 bp were trimmed and then assigned to soil samples based on unique 5-bp barcodes. After denoising and chimera filtering using usearch pipeline (Reeder and Knight, 2010; Haas et al., 2011), the quality reads were then binned into operational taxonomic units (OTUs) using a 97% identity threshold, and the most abundant sequence from each OTU was selected as the representative sequence. Taxonomy was then assigned to bacterial OTUs with reference to a subset of the SILVA 119 database^1^.

We obtained total 9,291,676 reads of bacterial 16S rRNA gene fragments, and between 65,778 and 226,631 reads per sample. A total of 3,640 OTUs were eventually obtained. For all OTU-based analyses, the original OTU table was rarified to a depth of 65,000 reads per sample to measure both diversity and species composition patterns, which was extremely close to the minimum sequence number for all samples.

### Calculation of Ecological Assembly Processes

Prior to null model construction, we first verified phylogenetic conservatism in our samples (Supplementary Figure S2) via testing the correlation coefficient relating between OTU phylogenetic distances to between-OTU niche distances (Stegen et al., 2013) using Mantel correlograms with 999 randomizations for significance tests (Warren et al., 2008; Diniz-Filho et al., 2010) with the function “mantel.correlog” in the R package vegan v2.0-2^2^. Then, we quantified the turnover of phylogenetic composition between samples (phylogenetic β-diversity) using between-community Mean Nearest Taxon Distance (βMNTD) and Nearest Taxon Index (βNTI) as per Stegen et al. (2012, 2013, 2015). βMNTD was calculated using the R function “comdistnt” (abundance.weighted = TRUE; package “picante”). The difference between observed βMNTD and mean of the null distribution of βMNTD generated by 1000 randomizations of the “phylogeny.pool” null model by its standard deviation is referred as βNTI. The values between −2 and +2 indicate the expectation under stochastic community assembly while the individual values below −2 or above +2 indicate that the observed difference in phylogenetic community composition is the result of deterministic selection (Stegen et al., 2013). We quantified these metrics for pairwise comparisons among 69 samples using any species present in a single sample as part of the regional species pool. This was done to specifically relate temporal dynamics of bacterial community composition to the soil pedogenesis and development. In addition, when we estimated patterns of ecological assembly processes with different successional timescales, phylogenetic null models were separately conducted by corresponding sub-datasets.

In addition to βNTI, the Bray-Curtis-based Raup-Crick (RC_bray_) null model was further used to quantify dispersal-based stochastic ecological processes generating the turnover of bacterial community composition (Stegen et al., 2015). RC_bray_ was based on a comparison between observed and expected levels of turnover, but without using phylogenetic information. The deviation between empirically observed Bray-Curtis and the null distribution was then standardized to vary between −1 and +1, and the resulting metric is referred to as RC_bray_. Values of RC_bray_ below −0.95 or above +0.95 indicate significant deviations from the null model expectation.

The relative contributions of ecological processes governing community turnover that are determined by homogeneous and variable selection are denoted by βNTI < −2 and βNTI > +2, respectively. The relative contributions of dispersal limitation and homogenizing dispersal processes are estimated by | βNTI| < 2 but RC_bray_ > +0.95 and RC_bray_ < −0.95, respectively. Furthermore, if | βNTI| < 2 and | RC_bray_| < 0.95, then undominated fraction drives compositional turnover process (Stegen et al., 2015).

### Identification of Bacterial Species With Time-Discriminatory Importance

To unravel taxa driving community changes across 630 years and/or uniquely characterizing successional eras (termed “biomarker taxa”), the relative abundances of bacterial taxa at the family level were regressed against successional eras using the function package “randomForest” of R software with default parameters. Biomarker taxa were identified using 10-fold cross-validation, implemented using R “rfcv” function.

### Statistical Analysis

Non-metric multidimensional scaling (NMDS) was performed to visualize the dissimilarities of bacterial community composition along the chronosequences based on Bray-Curtis (OTU level) and βMNTD (phylogenetic level) distances. Permutational multivariate analysis of variation (PERMANOVA) test was conducted to confirm the significance of dissimilarity. The above analyses were conducted using “vegan” package in R software (Vegan package, Version 3.1.2). Analysis of variance (ANOVA) was used to test the significance of variation in diversity indices among eras. These statistical procedures were conducted by SPSS16.0 (Statistical Product and Service Solutions). To unravel the drivers influencing community composition, taxonomic (Bray−Curtis) and phylogenetic (βMNTD) turnovers, as well as βNTI across and within different successional eras were regressed against soil chemical variables alone and in combination by Mantel test with 999 permutations. For all the statistical tests, significance and high significance were determined as *p* < 0.05 and *p* < 0.01, respectively.

## Results

### Bacterial Community Composition Across Longji Rice Terraces Cultivation History

**FIGURE 1:**
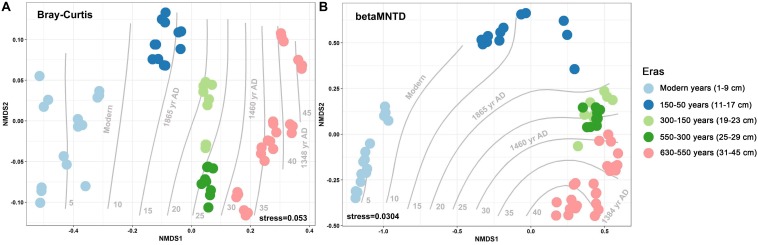
Non-metric multidimensional scaling (NMDS) with Bray–Curtis **(A)** and between-community mean-nearest-taxon-distance (βMNTD) **(B)** (*n* = 69) distances indicates the chronological succession of soil bacterial community along 630-year rice cultivation in Longji terraces.

**FIGURE 2:**
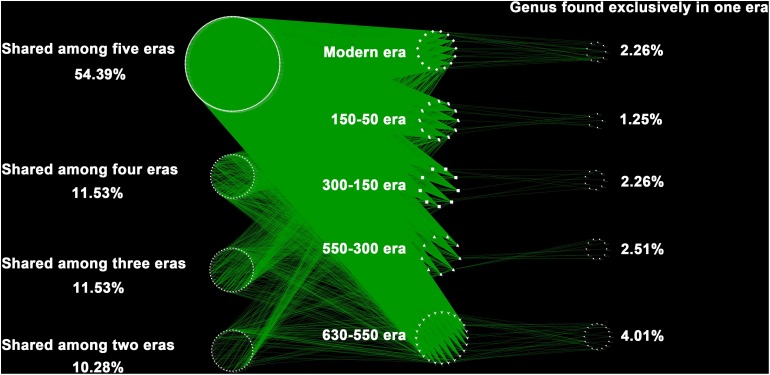
The distribution of the genera among eras. Each point represents one independent bacterial genus (414 genera in total). In the right column were unique to one era, whereas those in the left belonged to multiple eras. Of the 414 distinct bacterial genus observed, the majority (87.7%) were found shared by eras, and much higher than those unique to single era (12.3%).

Over the 630 years of cultivation, bacterial communities in Longji terraces have self-assembled into five groups (i.e., 1−9 cm, 11−17 cm, 19−23 cm, 25−29 cm, and 31−45 cm layers) as indicated by NMDS (Figure 1). This grouping represents their historical eras: modern (50-0 years), 150-50, 300-150, 550-300, and 630-550 years by radiocarbon dating (Jiang et al., 2014). Furthermore, microbial communities were more closely clustered within an era, and distinctly separated from other eras, except for smaller phylogenetic differences between 300-150 and 550-300 eras (PERMANOVA, Supplementary Table S1). This phenomenon indicated that the shifts of bacterial communities were along successional eras. Presumptively, we defined these groups as five chronological eras from here on in. Further, the distribution of the genera among eras revealed that 54.39% of generaa were shared among five eras: 11.53%, 11.53%, and 10.28% were shared among four, three and two eras; respectively; and 12.3% of genera were unique to single era (Figure 2). The results indicated that while there was a core microbial community across all eras, 45.61% of genera changed through time, denoting microbial succession and community shifts across 630 years.

**FIGURE 3:**
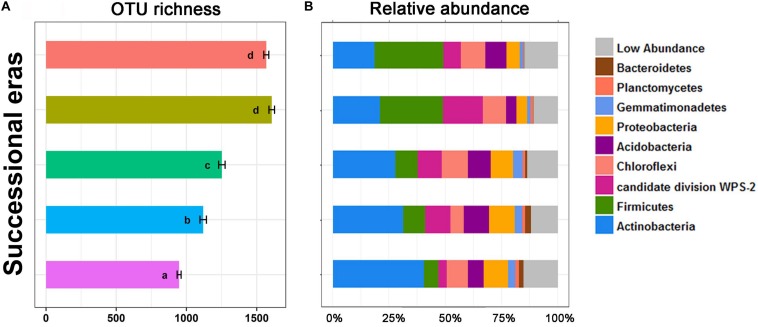
OTU richness **(A)** and stack plot of relative abundances of bacterial community composition **(B)** among five successional eras along the 630-year rice cultivation. Letters over error bar denote significant differences.

Alpha diversity and community composition were also observed to chronologically shift along five successional eras. OTU richness (Figure 3) was calculated to evaluate variation in bacterial alpha diversity throughout the chronological cultivation history. Detailed changes in patterns of diversity within layers are presented in Supplementary Figure S3. Overall, OTU richness increased along the cultivation history (*p* < 0.05), but no significant difference in OTU richness was observed between modern era and the 150-50 era (p > 0.05). Regardless of era, the bacterial community was dominated by several phyla, including Actinobacteria, Firmicutes, Chloroflexi, Proteobacteria, Acidobacteria, Gemmatimonadetes, Planctomycetes, and Bacteroidetes. Significant differences in bacterial community composition at phylum level were detected among five eras (Figure 3B). Amongst these, the greatest increase was on Firmicutes, from 6.45% in the 630-550 era to 30.73% in modern era (*p* < 0.01), while the largest decrease was on Actinobacteria, from 40.33% to 18.4% (*p* < 0.01).

### Influence of Environmental Variables on Taxonomic and Phylogenetic Community Composition

We evaluated the influence of different environment variables on bacterial alpha diversity (OTU richness) across 630 years of cultivation. Soil chemical properties of each successional era are presented in Supplementary Table S2. Positive correlations of alpha diversity with SOC, TN and TP contents were ubiquitously observed (*p* < 0.001), while correlations became negative with Fe, Mn, pH, Ca, Cu, K, Mg, and Zn values (Table 1). Additionally, we found significant correlations between soil chemical properties and community composition across 630 years of succession as well within each successional era were using Mantel tests (Table 2 and Supplementary Table S3) and Procrustes analysis (Supplementary Figure S4). In particular, strong correlations between Bray-Curtis dissimilarity and soil fertility (as the integrated proxy of TN, TP, Na, Ca, Cu, K, Mg, Zn, and SOC) were observed. Soil fertility showed the highest correlations with βMNTD across 630 years as well at modern, 150-50, and 630-550 eras. Conversely within the 550−150 era, soil redox conditions (Fe and Mn) were the most significant environmental factors associated with community composition.

**TABLE 1:** Correlations between alpha diversities as well as abundances and soil chemical variables.

	SOC	TN	TP	Fe	Mn	pH	Na	Ca	Cu	K	Mg	Zn
Observed OTU	0.642**	0.636**	0.634**	−0.704**	−0.414*	−0.776**	0.03	−0.496**	−0.406**	−0.41*	−0.652**	−0.352*
PD	0.623**	0.629**	0.625**	−0.712**	−0.412**	−0.773**	0.052	−0.57**	−0.421**	−0.305*	−0.400**	−0.620**
Chao1	0.624**	0.643**	0.638**	−0.693**	−0.376**	−0.783**	0.009	−0.521**	−0.399**	−0.374*	−0.625**	−0.335*
Shannon	0.565**	0.563**	0.546**	−0.697**	−0.4**	−0.638**	0.086	−0.556**	−0.374*	−0.344*	−0.606**	−0.337*
Abundance	0.882**	0.879**	0.791**	−0.772**	−0.743**	−0.640	−0.003	−0.082	−0.167	−0.47**	−0.538*	−0.455**

*Difference is at 0.05 level. **Difference is at 0.01 level.

**TABLE 2:** Mantel tests between Bray**−** Curtis dissimilarity as well as βMNTD distance and soil chemical properties across 630 years and within successional eras.

	Variables	Across 630 years	630−550 era	550−300 era	300−150 era	150−50 era	Modern era
Bray−Curtis	ENV-All	0.782**	0.576**	0.869**	0.845**	0.726**	0.861**
	ENV-Soil fertility	0.791**	0.591**	0.868**	0.831**	0.734**	0.863**
	ENV-Nutrient	0.765**	0.592**	0.862**	0.881**	0.695**	0.834**
	ENV-Redox	0.337**	0.320**	0.711**	0.623*	0.529**	0.551**
βMNTD	ENV-All	0.808**	0.522**	0.233**	0.721**	0.701**	0.551**
	ENV-Soil fertility	0.821**	0.528**	0.226**	0.612**	0.712**	0.553**
	ENV-Nutrient	0.783**	0.529**	0.191*	0.686**	0.678**	0.500**
	ENV-Redox	0.326**	0.361**	0.352**	0.779*	0.572**	0.398**

ENV-Nutrient includes TN, TP, Na, Ca, Cu, K, Mg, and Zn. ENV-Soil fertility includes nutrient and SOC. ENV-Redox includes Fe and Mn. *Difference is at 0.05 level. **Difference is at 0.01 level.

### Ecological Assembly Processes Governing Succession of Community Composition

**FIGURE 4:**
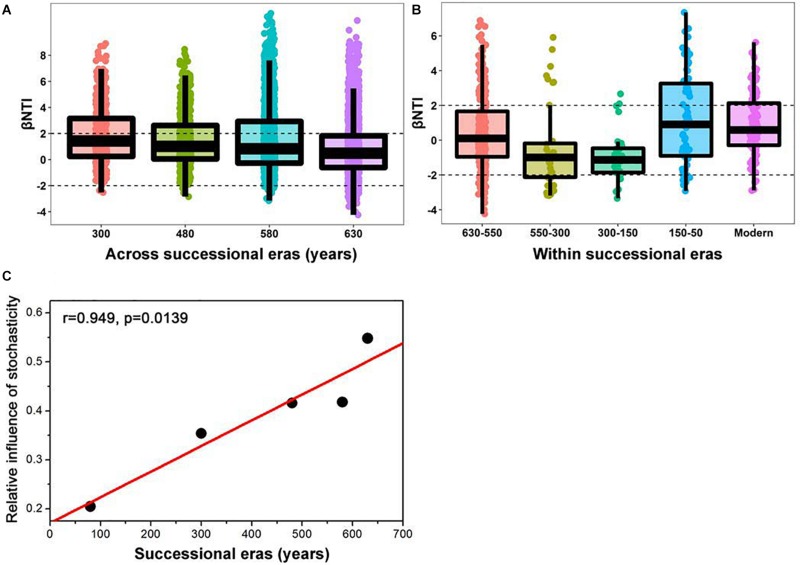
Patterns of βNTI across **(A)** and within **(B)** successional eras along 630-year of Longji chronosequences. Horizontal dashed lines indicate the βNTI significance thresholds of +2 and –2, respectively. The linear correlation between relative influence of stochasticity and successional eras **(C)**.

A linear shift in balance from deterministic to stochastic processes was observed as timescale increased, and the relative importance of ecological processes governing community assembly differed at shorter timescales within each succession era (Figure 4 and Table 3). Specifically, we observed increasing relative influence of stochasticity with increasing successional timescale [the initial 80 years (630-550 successional era), and across successional eras (300, 480, 580, and 630 years)] (*p* = 0.0139, Figure 4C). Deterministic processes were weak when investigating microbial assembly at long timescales (>300 year) and were uncorrelated with time (*p* = 0.1927); they accounted for less than 37.5% of microbial community assembly processes across successional eras. For example, across all 630 years, community assembly was mostly dominated by stochastic processes (54.7%), instead of deterministic processes (27.8%). Conversely, within successional eras (less than 250 years, e.g., 550−300 era), deterministic and stochastic processes alternatively governed community assembly (Figure 4B and Table 3). In particular, stochastic processes played important roles in both modern (67.6%) and 300-150 eras (37.1%). These two eras were primarily governed by dispersal limitation (58.1%) and homogenizing dispersal (37.1%), respectively. In contrast, relatively strong deterministic processes, dominated by variable selection, were found for the 150-50 (51.5%) and 630-550 (35.9%) eras.

**TABLE 3:** The relative contributions of ecological assembly processes across and within successional eras.

		Variable selection	Homogeneous selection	Deterministic	Dispersal limitation	Homogenizing dispersal	Stochastic	Undominated
Across	300 years (630-300)	0.371	0.004	0.375	0.311	0.044	0.354	0.259
	480 years (630-150)	0.311	0.003	0.314	0.382	0.033	0.416	0.254
	580 years (630-50)	0.338	0.008	0.347	0.379	0.038	0.418	0.229
	630 years (630-Modern)	0.232	0.047	0.278	0.492	0.056	0.547	0.174
Within	630-550 era	0.231	0.128	0.359	0	0.205	0.205	0.436
	550-300 era	0.222	0.278	0.500	0	0.500	0.500	0
	300-150 era	0.057	0.200	0.257	0	0.371	0.371	0.371
	150-50 era	0.364	0.152	0.515	0.212	0.061	0.273	0.212
	Modern era	0.276	0.038	0.314	0.581	0.095	0.676	0.010

*Deterministic assembly processes = Variable selection + Homogeneous selection; Stochastic assembly processes = Dispersal limitation + Homogenizing dispersal.*

### Environment Variables Influencing Community Assembly Through Time

To understand the key environmental variables responsible for community assembly along a successional gradient, correlations between βNTI values and change in environmental variables were conducted for all possible pairs across and within successional eras (Table 4 and Supplementary Table S4). When investigating assembly over 300 years or more (including ca. 300, 450, 550 and 630 years), almost no strong correlations were found, as all Mantel test coefficients were less than 0.3. However, within-era associations between βNTI and environmental variables differed across successions. Specifically, soil fertility showed the most important correlation with βNTI for 150-50 (*r* = 0.577, *p* < 0.01) and 630-550 (*r* = 0.445, *p* < 0.01) eras, and soil redox conditions presented a significant influence on 300-150 era (*r* = 0.661, *p* < 0.01). Both fertility and redox conditions were important drivers for modern era (*r* = 0.313, *p* < 0.01 vs. *r* = 0.312, *p* < 0.01). Similar to the results of over 300 years, no significant correlations between chemical variables and βNTI were found for 550-300 era.

**TABLE 4:** Mantel tests between βNTI and soil chemical properties across and within successional eras.

		ENV-All	ENV-Soil fertility	ENV-Nutrient	ENV-Redox
Across	300 years (630-300)	0.274**	0.283**	0.255**	0.153*
	480 years (630-150)	0.218**	0.227**	0.205**	0.108
	580 years (630-50)	0.024	0.035	0.011	−0.05
	630 years (630-Modern)	0.026	0.033	0.023	−0.564
Within	630-550 era	0.440**	0.445**	0.444**	0.307**
	550-300 era	0.031	0.0277	0.005	0.127
	300-150 era	0.486**	0.36*	0.398*	0.661**
	150-50 era	0.563**	0.577**	0.547**	0.451*
	Modern era	0.315**	0.313**	0.297**	0.312**

ENV-Nutrient includes TN, TP, Na, Ca, Cu, K, Mg, and Zn. ENV-Soil fertility includes nutrient and SOC. ENV-Redox includes Fe and Mn. *Difference is at 0.05 level. **Difference is at 0.01 level.

### Biomarker Bacterial Species With Time-Discriminatory Importance

**FIGURE 5:**
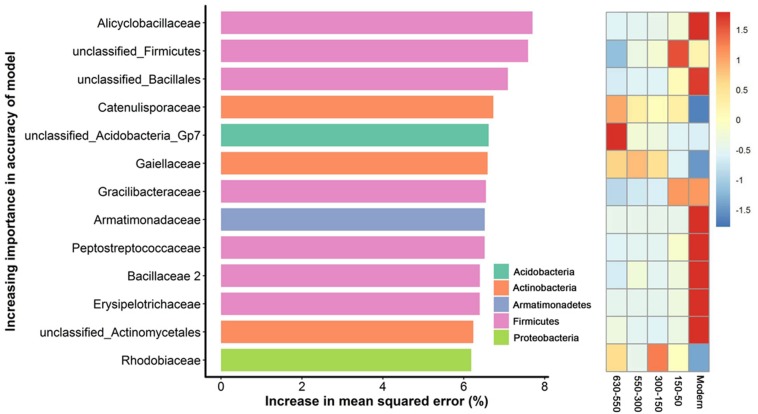
Bacterial taxonomic biomarkers of within successional eras. The top 13 biomarker taxa were ranked in descending order of time-discriminatory importance to the accuracy of the models. Heatmap showed the variations in the relative abundances of these top 13 predictive biomarker taxa among successional eras.

To unravel the biomarker species discriminating bacterial communities along successional eras of rice cultivation, we performed a regression of the relative abundances of bacterial species at the family level against five successional eras using the random forest machine learning algorithm. The minimum cross-validation error was obtained when using 205 important families. The top 13 most important families were chosen as the respective biomarker taxa because cross-validation error curve had stabilized when using these families (Figure 5). The most important biomarker species for the time discrimination were classified into phyla of Acidobacteria, Actinobacteria, Armatimonadetes, Firmicutes and Proteobacteria. Generally, Firmicutes played important roles in modern era with Alicyclobacillus of the highest value, while phototrophic bacteria (i.e., Rhodobiaceae) were important within 300-150 era.

## Discussion

### Archived Microbial Information in Longji Rice Paddies

We used chronosequential soils at Longji rice paddies to unravel temporal patterns in microbial community assembly in a sustainable agroecosystem. It was first necessary to confirm that the microbial DNA archived in each soil horizon was an appropriate proxy for soil successional era. NMDS plots based on taxonomic and phylogenetic composition depicted clear shifts in bacterial community along the cultivation chronosequence (Figure 1). Mantel tests and Procrustes analysis further demonstrated significant correlations between soil chemical properties and bacterial community composition across successional eras (Table 2, Supplementary Figure S4, and Supplementary Table S4). Buried microorganisms can persist over long periods of time in a dormant state (Demkina et al., 2000; Mitusov et al., 2009) and, in palaeosols, are characterized by a slow metabolism with sparse use of external energy sources at a low water potential (Brockman et al., 1992; Demkina et al., 2007). Thus, using cultivable dependent method, Khomutova et al. (2006), Grund et al. (2014) found that viable microbial information can well reflect the archeological information. In previous investigations, Methanocellaceae, organisms closely associated with rice growth (Lu and Conrad, 2005), have been verified to drastically decrease in abundance with Mn content in Longji paddy soils (Jiang et al., 2014), and that both Methanocellaceae and Mn content supported the reconstruction of the influence of historical events on rice cultivation (Feng et al., 2017a). If the majority of microorganisms were active in palaeosols, their metabolism would inevitably influence patterns of elements along depth profile. Thus, the dramatic shifts in Methanocellaceae abundance and Mn content would be not observed along profile, as one would expect smoother changes in microorganisms and elements along a typical redox gradient. For the same reason, when linking several distinct changes in bacterial community revealed by GradientForest model with the turning points of SOC content (as the proxy of soil fertility) due to historical events in 630 years in this investigation, it was found that their changes coincided with each other (Supplementary Figure S5). Furthermore, extracellular microbial DNA can also be preserved in palaeosols for thousands of years (Rawlence et al., 2014). Archived microbial DNA (both intra- and extracellular) in palaeosols had been extensively applied into microbial ecological investigations (Zhu et al., 2016; Ding et al., 2017; Feng et al., 2017a). Putting these findings and statements together, we believe that soil chronosequences of Longji terraces offer an ideal opportunity for investigating temporal community assembly in successional soils.

Changes related to physicochemistry (e.g., O_2_ and nutrients) along depth profiles influenced microbial communities (Eilers et al., 2012; Jiao et al., 2018). We admitted that the general changing patterns of our microbial data are somewhat similar to those investigations on depth profile (Grund et al., 2014; Wurzbacher et al., 2017). It means that the current perturbations on microbial community archived into the soil profile cannot be ruled out. While in our manuscript, several pieces of evidence suggested that the occurrence of community variations coincided with associated environmental thresholds associated with successional eras rather than continuous environmental gradients, which supports our assumption that paleosols maintained the microbial communities that existed when they were buried to a great degree. Taken together, we suggested that the historical microbial information can be well archived in the soil profile of Longji terraced paddy, although the effects of current perturbation also occurred. In the future, these two effects should be disentangled to comprehensively unravel temporal assembly mechanisms in Longji terraced soil profiles.

### The Balance Between Deterministic and Stochastic Processes Governs Community Assembly Within Successional Eras (∼Decadal Scale)

Within successional eras, stochastic processes played important roles in both 300-150 (37.1%) and modern (67.6%) eras, possibly due to cultural and land use practices that promoted spatial isolation or enhanced hydrologic mixing in each period. In modern era soils, stochastic processes were primarily attributed to dispersal limitation (Table 3). Modern fertilization has been shown to increase the percentage of macro-aggregates in soil, as well as their stability (Tang et al., 2018; Zhang et al., 2018). The stability and content of soil aggregates are positively correlated with soil fertility because the cohesion of aggregates is promoted mainly by organic polymer binding agents (Zhang et al., 2018). By forming soil aggregates, organic matter is less susceptible to be consumed by soil microorganisms due to spatial isolation (Schmidt et al., 2011). Because of this physical disconnection, each aggregate may also represent a different ecological niche for microbial colonization (Trivedi et al., 2015). In modern era soils, it is reasonable to deduce that microbes are largely contained in each macro-aggregate, and their dispersal processes are constrained. In contrast, within 550-150 era soils, homogenizing dispersal dominated stochastic processes. South China entered the Little Ice Age during this era (Ge et al., 2011). Such environmental conditions are speculated to impact human farming activities and weaken hydroponic farming activities (Jiang et al., 2014). Consequently, paddy soils are thought to have been transported to upland farming or even abandoned, disrupting the clear paddy soil compartments system formed by rice cultivation. Therefore, hydrologic mixing associated with land use change likely facilitated high rates of homogenizing dispersal during this period.

We also found that deterministic assembly processes in all successional eras were governed by soil fertility and/or redox conditions linked to anthropogenic land use practices (Table 4). Though all successional eras exhibited some influence of determinism, relatively strong deterministic processes were found for the 630-550 (35.9%) and 150-50 (51.5%) soil eras, which were both dominated by variable selection (23.1% and 36.4%). Soil redox conditions are associated with the presence-absence of oxygen and reflected by Fe and Mn content (Chen et al., 2011; Byrne et al., 2015; Jiang et al., 2017). During the 630-550 era, a major shift in land use created the assart of paddy cultivation. Similarly, within 300-150 era (namely after Little Ice Age), paddy cultivation gradually recovered from adverse palaeoclimate conditions (Jiang et al., 2014). Both eras experienced a huge transformation between upland and hydroponic systems during which the oxygen partial pressure in paddy soils changed dramatically, and Fe and Mn content were most significant correlated with βNTI during these eras (Table 4).

In addition to redox conditions, soil fertility had the greatest effect on assembly processes in eras that showed the highest impact of determinism (630-550 and 150-50 eras) (Table 4). These eras constituted the early and stable periods of paddy reclamation, respectively, and thus soil fertility was continuously enhanced during these periods (Jiang et al., 2014). We have previously shown that an increase in soil fertility can stimulate specific microorganisms and impose a strong directional change in composition (Feng et al., 2015). Our finding that deterministic assembly processes were strongest during time periods with dramatic increases in fertility aligns with this observation and with other work showing that changes in soil fertility deterministically influence community composition (Dini-Andreote et al., 2015; Liu et al., 2015b; Stegen et al., 2016; Zhang et al., 2016). We also note that rice paddies are compartmentalized systems in which dissimilar physiochemical conditions can persist in different compartments and in which well-defined microscale chemical gradients exist due to the presence and absence of oxygen (Liesack et al., 2000). These delineated environments would have contributed to the variable selective pressures we observed.

Soil pH can be one of the major factors that influence ecological assembly processes, especially in systems with physical disturbances or land use changes that create drastic variations in pH (Ferrenberg et al., 2013; Ding et al., 2017; Tripathi et al., 2018), however, we found little impact of pH on microbial community assembly in our study (Supplementary Table S4). We suggest that the subtle variation in pH we observed is not large enough to drive the turnover of community composition during succession of paddy soils. In contrast, owing to the history of anthropogenic agricultural activities over 630 years and the particular profile of pedogenesis characteristics, soil fertility and soil redox conditions overrode any influence of pH in this case.

### A Directional Shift in Balance From Deterministic to Stochastic Processes Governs Community Assembly Across Successional Eras (∼Centurial Scale)

In contrast to patterns within successional eras, assembly processes across successional eras were gradually governed by stochastic processes (Table 3). In other words, a linear shift towards stochasticity with increasing timescale was observed (Figure 4C). It should be noted that successional eras co-varied with soil fertility (*r* = 0.851, *p* < 0.01) and redox conditions (*r* = 0.592, *p* < 0.01) in this case, implying that niche variations could also influence long-term patterns of assembly processes. Our results are inconsistent with studies investigating microbial assembly following wildfire disturbance (Ferrenberg et al., 2013), glacial retreat (Tripathi et al., 2018), and salt marsh intrusion (Dini-Andreote et al., 2015), however, our work examined drastically longer timescales (centuries) than these prior investigations (months to decades). The above works consistently indicated that stochastic processes governed the community assembly during early succession, and the relative importance of deterministic processes increased in as succession progressed. Our phenomenon might be ascribed to unique features of rice cultivation and the longer timescale of our investigation. Long-term rice cultivation promotes soil fertility (Chen et al., 2011), which leads to more bioavailable energy and nutrients that release microorganisms from environmental stresses due to resource limitation (Feng et al., 2017b, 2018). In addition, flooding can help paddy soils temper extremes in environmental changes and increase putative stochastic dispersal process. Previous work has demonstrated that stochastic processes play a heightened role when environmental stressors are low (Stegen et al., 2012). We therefore propose that gradual environmental changes of paddy soils lead to stochasticity as the dominant assembly processes at longer (centurial) timescales. In turn, stochasticity promotes biodiversity that generates a positive feedback for sustainable ecosystem function (Graham and Stegen, 2017).

### Implications of Microbial Ecological Assembly Processes for Paddy Soil Pedogenesis and Agroecosystem Sustainability

**FIGURE 6:**
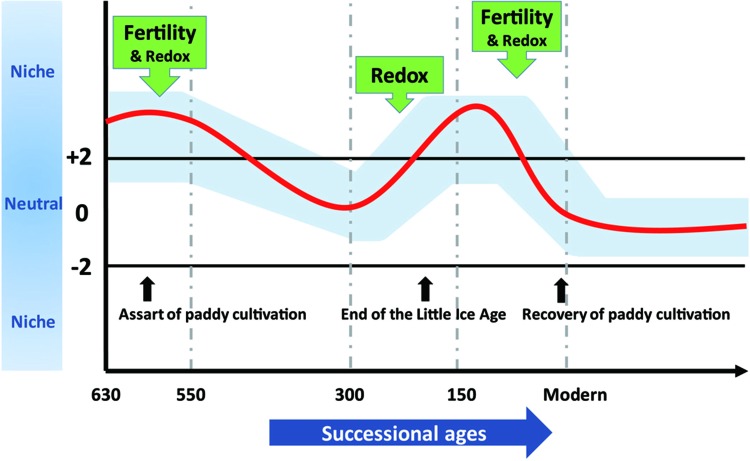
The assembly processes governing bacterial community structure along 630-year rice cultivation. The whole 630-year succession of bacterial communities appeared to be governed by stochastic processes, whereas stochastic and deterministic assembly processes jointly governed microbial community structure within successional eras, and within-era determinism was mainly driven by soil fertility and redox conditions alone or in combination. We propose that these dynamics underlie sustainable and efficient ecological functions of paddy ecosystem and highlight the importance of time dependence in microbial community assembly.

To demonstrate effects of assembly processes on agroecosystem sustainability at various timescales, we synthesized our results into a conceptual model depicted in Figure 6. We propose that long-term stochasticity combines with short-term variability in assembly processes to underlie microbial succession in rice paddies. Changes in community assembly broadly influence microbial composition (Graham et al., 2016) and microorganisms in stochastically assembled communities may offer more diversified functional traits that promote ecosystem sustainability (Chase, 2010; Deng et al., 2016; Soliveres et al., 2016). Contrarily, determinism can favor particular microorganisms that function at high levels in optimized environments (e.g., fertility or redox status) (Knelman and Nemergut, 2014; Graham et al., 2016; Graham and Stegen, 2017). The fluctuation between stochastic and deterministic processes through time not only guarantees multifunctionality of microorganisms but also preserves particular traits in paddy soils that may dominate under certain circumstances.

Here, we identified diverse biomarker species (i.e., Acidobacteria, Actinobacteria, Bacteroidetes, Candidatus Saccharibacteria, Firmicutes and Proteo-bacteria) at stochastic processes dominated 630-year rice cultivation (Figure 5) that may support multifunctionality in microbial communities. In contrast, specific biomarker taxa differed across eras structured by determinism. For instance, Rhodobiaceae became dominant biomarker species in 300-150 eras, when land use changes deterministically impacted rice paddy cultivation, putatively by promoting the growth of facultative anaerobic microorganisms that thrive in the transition zone between oxic and anoxic conditions. Rhodobiaceae are facultative anaerobic microbes, and they are widely distributed in paddy soils with rice-wheat rotation systems (Feng et al., 2011). Additionally, Bacillus, which is known to respond to increases in soil fertility (Feng et al., 2015), was an indicator taxon for fertility accumulated in Modern eras. Alicyclobacillus in particular was the most important biomarker in modern era of this study and has been previously linked to phosphorus (Long et al., 2018), iron (Yahya et al., 2008) and sulfur (Guo et al., 2009) cycling. These dynamics may therefore underpin sustainable and efficient ecological functions of paddy ecosystem that are often observed (Branch et al., 2007; Chen et al., 2011; Zhu et al., 2016; Feng et al., 2017a).

## Conclusion

We find that deterministic and stochastic processes simultaneously govern community assembly through successional time but that the influence of each varies both with successional era and with the duration of the time period examined. This finding highlights the importance of time dependence in microbial community assembly. Zhang et al. (2016) posited that the deterministic processes are important over longer timescales while stochastic processes govern short-term assembly processes; however, our findings are contrary to this suggestion. Long-term (630-years) community assembly was primarily stochastic, and the relative importance of stochasticity and determinism fluctuated at shorter timescales (i.e., within each successional era). Our work therefore provides an insight on long-term mechanisms driving microbial community composition than are typically considered and informs microbial changes over decadal-to-centurial timescales that are vital to maintaining agroecosystem sustainability.

## Data Availability Statement

The sequences were deposited in NCBI SRA database (Accession No. SRP158720).

## Author Contributions

XL, SL, and YF designed the experiments. WL, JZ, and YF performed the experiments. WL, EG, JZ, LZ, and YF analyzed the data. All authors contributed to data interpretation and writing of the manuscript.

## Conflict of Interest

The authors declare that the research was conducted in the absence of any commercial or financial relationships that could be construed as a potential conflict of interest.

## Footnotes

1 http://www.arb-silva.de/download/archive/qiime/

2 http://vegan.r-forge.r-project.org
