# Supplementary material for: Long-Term Stochasticity Combines With Short-Term Variability in Assembly Processes to Underlie Rice Paddy Sustainability
Source: Front Microbiol. 2020 May 15;11:873. doi: 10.3389/fmicb.2020.00873 (PMC7243440; doi:10.3389/fmicb.2020.00873)
Supplement: Supplementary file 1 [file Data_Sheet_1.docx]

**Supplementary information**

**Table S1.** Dissimilarity tests of bacterial communities among five eras using Permutational multivariate analysis

based on Bray-Curtis dissimilarity and βMNTD distance.

| **PERMANOVA test** | **Bray-Curtis** | |  | **βMNTD** | |
| --- | --- | --- | --- | --- | --- |
|  | **r^2^** | **p.value** |  | **r^2^** | **p.value** |
| **Modern vs 150-50** | 0.624 | 0.001 |  | 0.511 | 0.001 |
| **Modern vs 300-150** | 0.695 | 0.001 |  | 0.794 | 0.001 |
| **Modern vs 550-300** | 0.698 | 0.001 |  | 0.807 | 0.001 |
| **Modern vs 630-550** | 0.679 | 0.001 |  | 0.883 | 0.001 |
| **150-50 vs 300-150** | 0.539 | 0.001 |  | 0.228 | 0.001 |
| **150-50 vs 550-300** | 0.653 | 0.001 |  | 0.370 | 0.002 |
| **150-50 vs 630-550** | 0.565 | 0.001 |  | 0.463 | 0.001 |
| **300-150 vs 550-300** | 0.409 | 0.001 |  | 0.059 | 1 |
| **300-150 vs 630-500** | 0.375 | 0.001 |  | 0.229 | 0.001 |
| **550-300 vs 630-550** | 0.351 | 0.001 |  | 0.240 | 0.001 |

**Table S2.** Changes in soil chemical variables of the investigated soil chronosequences among different successional eras.

|  | **630-550 era** | **550-300 era** | **300-150 era** | **150-50 era** | **Modern era** |
| --- | --- | --- | --- | --- | --- |
| **SOC (g/kg)** | 10.74±1.82a | 12.59±0.69b | 10.75±0.76a | 12.97±1.13b | 20.45±2.20c |
| **TN (g/kg)** | 1.05±0.15a | 1.22±0.06ab | 1.05±0.06a | 1.26±0.12b | 2.01±0.22c |
| **TP (g/kg)** | 0.30±0.09ab | 0.32±0.02ab | 0.29±0.02a | 0.39±0.03b | 0.61±0.09c |
| **Fe (mg/g)** | 20.72±0.97c | 19.03±0.22b | 19.73±0.05b | 19.17±0.18b | 17.22±0.48a |
| **Mn (mg/g)** | 313.01±41.70c | 287.29±8.72abc | 305.38±39.26bc | 277.54±11.26ab | 269.97±5.02a |
| **pH** | 4.64±0.07c | 4.44±0.07b | 4.44±0.06b | 4.20±0.21a | 4.32±0.14ab |
| **Na (mg/g)** | 0.525±0.04a | 0.56±0.02ab | 0.589±0.01b | 0.524±0.05a | 0.534±0.02a |
| **Ca (mg/g)** | 0.287±0.04a | 0.275±0.02a | 0.251±0.03a | 0.249±0.03a | 0.260±0.03a |
| **Cu (mg/g)** | 15.17±0.12c | 14.58±0.07ab | 14.30±0.76ab | 13.70±0.97a | 14.51±0.67ab |
| **K (mg/g)** | 22.23±3.97b | 20.27±0.57ab | 21.40±1.14b | 20.21±1.43ab | 17.76±0.78a |
| **Mg (mg/g)** | 3.72±0.38c | 3.43±0.13b | 3.40±0.09b | 3.30±0.15ab | 3.01±0.13a |
| **Zn (mg/g)** | 71.36±11.43bc | 61.43±1.03a | 75.24±4.50c | 65.92±0.57ab | 58.90±4.23a |

^Values are means ± standard deviation. Different letters within the same column denote significant differences (p < 0.05) among soils.^

**Table S3.** Mantel tests between Bray-Curtis dissimilarity, βMNTD distance and soil chemical variables across 630 years and within successional eras.

|  | **Variables** | **Across 630 years** | **630-550 era** | **550-300 era** | **300-150 era** | **150-50 era** | **Modern era** |
| --- | --- | --- | --- | --- | --- | --- | --- |
| **Bray-Curtis** | SOC | 0.769** | 0.525** | 0.611** | 0.258* | 0.898** | 0.772** |
|  | TN | 0.766** | 0.496** | 0.704** | 0.16 | 0.879** | 0.779** |
|  | TP | 0.727** | 0.512** | 0.546* | 0.550* | 0.503** | 0.524** |
|  | Fe | 0.711** | 0.354** | 0.832** | 0.508* | 0.371* | 0.486** |
|  | Mn | 0.285** | 0.274** | 0.637** | 0.621* | 0.495** | 0.576** |
|  | pH | 0.34** | 0.013 | 0.423* | 0.772** | 0.262* | 0.516** |
|  | Na | 0.086* | 0.318** | 0.590* | 0.717** | 0.530** | 0.526** |
|  | Ca | 0.238** | 0.449** | 0.707** | 0.793** | 0.534** | 0.554** |
|  | Cu | 0.08 | 0.377** | 0.663** | 0.836** | 0.859** | 0.690** |
|  | K | 0.516** | 0.273** | 0.617** | 0.614* | 0.521** | 0.736** |
|  | Mg | 0.490** | 0.238** | 0.709** | 0.172 | 0.462* | 0.326** |
|  | Zn | 0.155* | 0.269** | 0.826** | 0.447* | 0.359* | 0.440** |
| **βMNTD** | SOC | 0.864* | 0.492** | 0.352* | -0.003 | 0.847** | 0.644** |
|  | TN | 0.862** | 0.484** | 0.356** | -0.05 | 0.841** | 0.605** |
|  | TP | 0.767** | 0.450** | -0.02 | 0.531* | 0.489** | 0.322* |
|  | Fe | 0.679** | 0.387** | 0.304** | 0.456* | 0.306* | 0.378** |
|  | Mn | 0.039** | 0.324* | 0.355* | 0.781* | 0.555** | 0.370** |
|  | pH | 0.143** | -0.2 | -0.1 | 0.866** | 0.166 | 0.292* |
|  | Na | 0.088* | -0.01 | 0.048 | 0.333* | 0.550** | 0.348** |
|  | Ca | 0.121* | 0.443** | 0.136 | 0.494* | 0.516** | 0.248* |
|  | Cu | -0.071 | 0.272** | 0.023 | 0.886** | 0.829** | 0.626** |
|  | K | 0.211* | 0.356** | 0.034 | 0.786** | 0.589** | 0.599** |
|  | Mg | 0.316** | 0.337** | 0.303* | -0.03 | 0.428* | -0.1 |
|  | Zn | 0.104* | 0.266* | 0.264** | 0.353* | 0.189 | 0.196* |

^**Difference is at 0.01 level. *Difference is at 0.05 level.^

**Table S4.** Mantel tests between βNTI and soil chemical variables across and within successional eras.

|  |  | SOC | TN | TP | Fe | Mn | pH | Na | Ca | Cu | K | Mg | Zn |
| --- | --- | --- | --- | --- | --- | --- | --- | --- | --- | --- | --- | --- | --- |
| **Across** | **300 years (630-300)** | 0.368** | 0.367** | 0.21** | 0.29** | 0.076 | 0.055 | 0.037 | 0.088 | 0.134 | 0.064 | 0.066 | 0.085 |
|  | **480 years (630-150)** | 0.266** | 0.277** | 0.176** | 0.275** | 0.035 | 0.031 | 0.063 | 0.058 | 0.067 | 0.027 | 0.038 | 0.016 |
|  | **580 years (630-50)** | 0.170** | 0.174** | 0.093 | -0.074 | -0.038 | -0.1 | -0.064 | -0.095 | -0.1 | 0.003 | -0.035 | -0.012 |
|  | **630 years (630-Modern)** | 0.053** | 0.055** | 0.053** | -0.045* | -0.045 | -0.099 | -0.057 | -0.0722 | -0.085 | -0.007* | -0.017* | -0.004 |
| **Within** | **630-550 era** | 0.053** | 0.055** | 0.053** | -0.045* | -0.045 | -0.099 | -0.057 | -0.0722 | -0.085 | -0.007* | -0.017* | -0.004 |
|  | **550-300 era** | 0.144 | 0.131 | -0.078 | 0.071 | 0.141 | -0.11 | -0.06 | 0.004 | -0.09 | -0.05 | 0.101 | 0.043 |
|  | **300-150 era** | 0.055 | 0.074 | 0.513** | 0.472* | 0.661* | 0.622* | -0.02 | 0.001 | 0.590** | 0.659** | 0.087 | 0.397* |
|  | **150-50 era** | 0.686** | 0.676** | 0.352* | 0.136 | 0.447** | 0.048 | 0.477* | 0.431** | 0.703** | 0.456* | 0.261* | 0.175 |
|  | **Modern era** | 0.280** | 0.249* | 0.270* | 0.290** | 0.325** | 0.219* | 0.554** | -0.062 | 0.437** | 0.315** | 0.008 | 0.334** |

^**Difference is at 0.01 level. *Difference is at 0.05 level.^

**
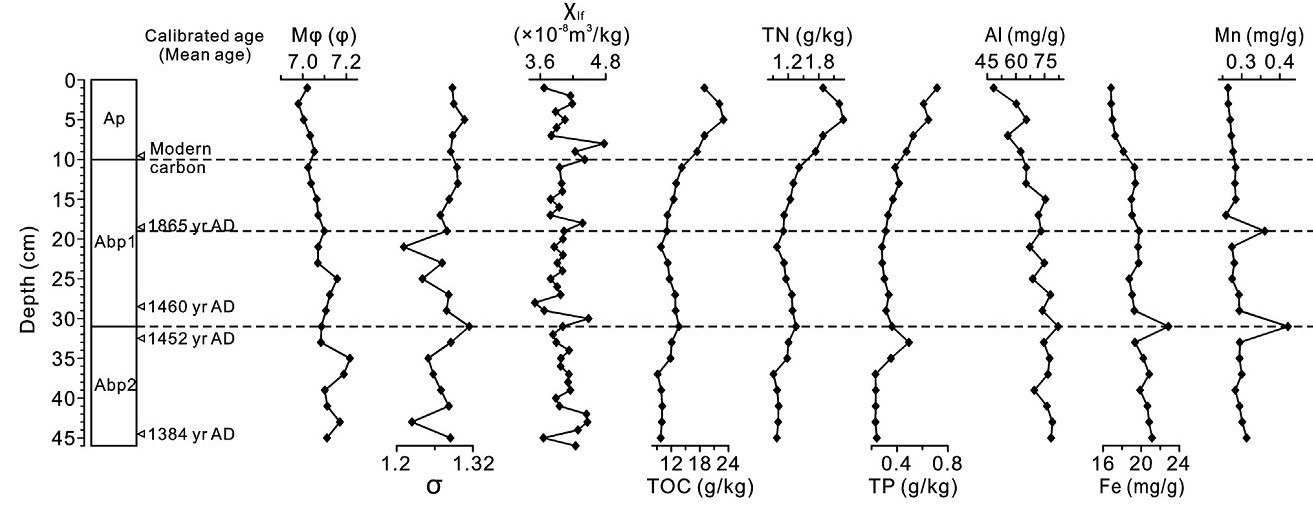
**

**Figure S1.** Data of physical and geochemical proxies from the cultivated horizon of profile LJTT-3 ([Jiang et al., 2014](#_ENREF_1)). Abbreviations: M_φ_, mean particle size; σ, sorting coefficient; X_lf_, low frequency mass-specific magnetic susceptibility.


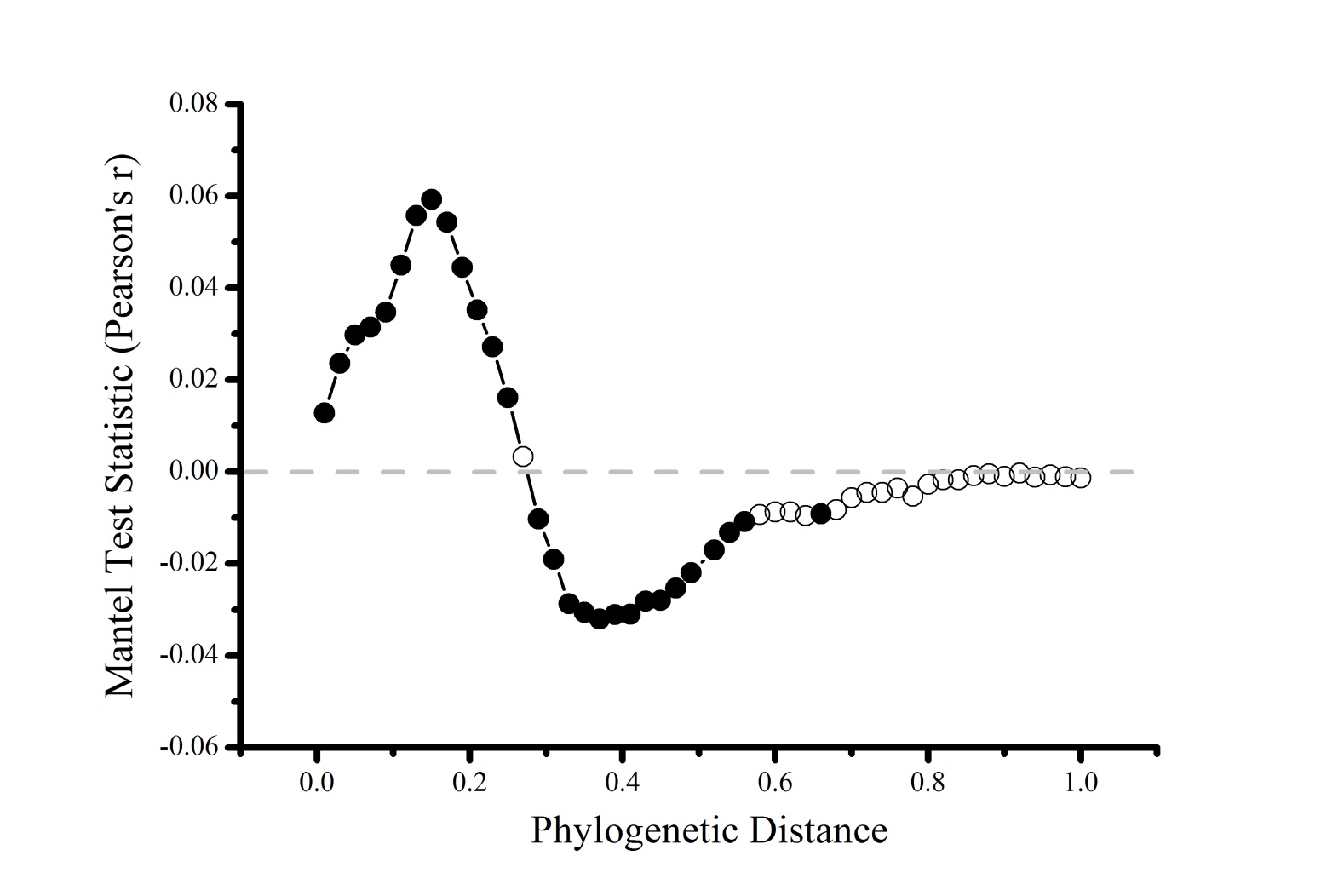


**Figure S2.** Phylogenetic mantel correlogram showing significant phylogenetic signal across short phylogenetic distances. The phylogenetic distance across which there was significant positive phylogenetic signal—whereby more closely related taxa are more ecologically similar—varied from 0% to 26% of the maximum phylogenetic distance. This suggests that bacterial ecological preferences are most strongly phylogenetically conserved across short phylogenetic distances. Ecological inferences can, therefore, be made from estimates of phylogenetic turnover ([Stegen, Lin, Fredrickson, & Konopka, 2015](#_ENREF_2); [Stegen, Lin, Konopka, & Fredrickson, 2012](#_ENREF_3)). Solid and open symbols denote significant (*p*<0.05) and insignificant (*p*>0.05) correlations, respectively, relating between-OTU niche differences to between-OTU phylogenetic distances within a given range of phylogenetic distances.

**
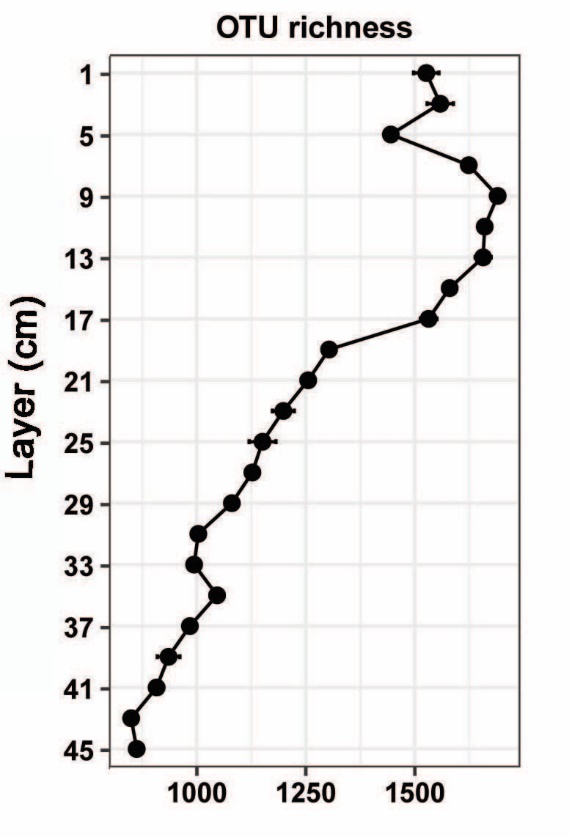
**

**Figure S3.** The variations of OTU richness within each layer.


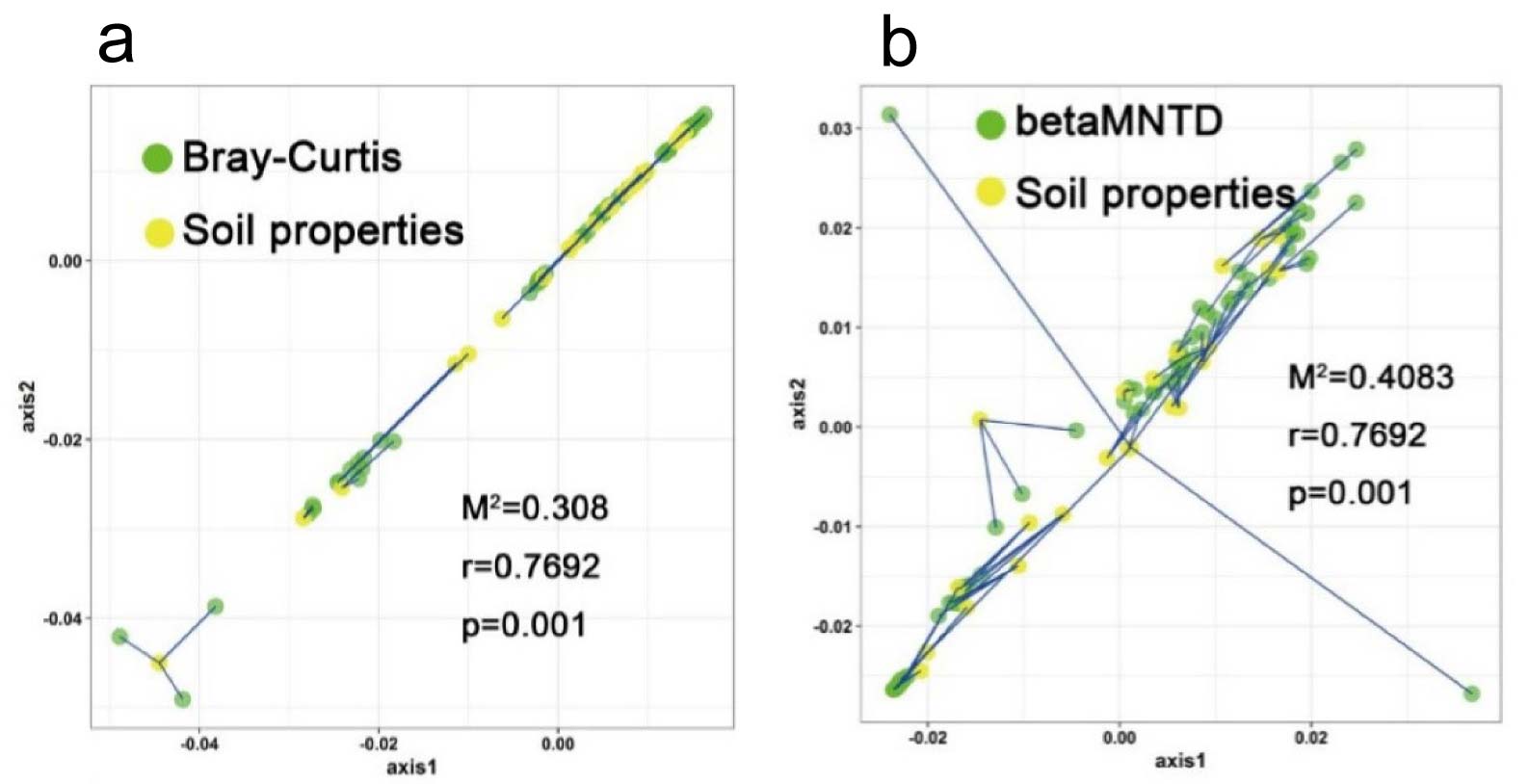


**Figure S4.** Procrustes analysis of significant correlations between soil properties distance and bacterial community composition based on Bray-Curtis dissimilarity (a) and βMNTD distance (b) respectively. The correlation M^2^ and r represent sum of square deviations and correlation coefficient, respectively. Soil properties consist of the SOC, TN, TP, Fe, Mn, Na, Ca, Cu, K, Mg, Zn, Al and pH.

**
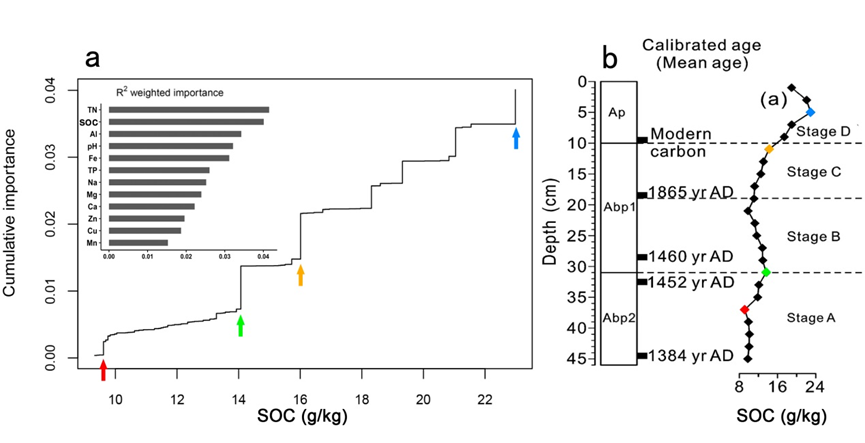
**

**Figure S5.** The cumulative compositional variations of bacterial community occurred at the key changing points of SOC, around 9, 14, 16 and 23 g kg^-1^, revealed by GradientForest model (Fig. S3 in (Jiang, Li et al. 2014)). The inset is the predictor importance plot illustrating the mean importance of each variable weighted by species R^2^.

Reference

Jiang, Y.J., Li, S.J., Cai, D.S., Chen, W., Liu, Y., & Yu, Z. (2014). The genesis and paleoenvironmental records of Longji agricultural terraces, southern China: A pilot study of human-environment interaction. *Quaternary International*, *321*, 12-21.

Stegen, J.C., Lin, X.J., Fredrickson, J.K., & Konopka, A.E. (2015). Estimating and mapping ecological processes influencing microbial community assembly. *Frontiers in Microbiology*, *6*.

Stegen, J.C., Lin, X.J., Konopka, A.E., & Fredrickson, J.K. (2012). Stochastic and deterministic assembly processes in subsurface microbial communities. *ISME Journal*, *6*, 1653-1664.
